# NEK7-Mediated Activation of NLRP3 Inflammasome Is Coordinated by Potassium Efflux/Syk/JNK Signaling During *Staphylococcus aureus* Infection

**DOI:** 10.3389/fimmu.2021.747370

**Published:** 2021-09-16

**Authors:** Ruiqing Liu, Yashan Liu, Chang Liu, Aijiao Gao, Lin Wang, Huixin Tang, Qiang Wu, Xia Wang, Derun Tian, Zhi Qi, Yanna Shen

**Affiliations:** ^1^School of Medical Laboratory, Tianjin Medical University, Tianjin, China; ^2^Key Laboratory of Emergency and Trauma, Ministry of Education, College of Emergency and Trauma, Hainan Medical University, Haikou, China; ^3^Department of Laboratory Medicine, West China Second University Hospital, Sichuan University, Key Laboratory of Birth Defects and Related Diseases Of Women and Children (Sichuan University), Ministry of Education, Chengdu, China; ^4^Department of Molecular Pharmacology, School of Medicine, Nankai University, Tianjin, China

**Keywords:** Syk, JNK, NLRP3 inflammasome, *S. aureus*, K^+^ efflux, NEK7

## Abstract

*Staphylococcus aureus* (*S. aureus*) is a foodborne pathogen that causes severe diseases, such as endocarditis, sepsis, and bacteremia. As an important component of innate immune system, the NLR family pyrin domain-containing 3 (NLRP3) inflammasome plays a critical role in defense against pathogen infection. However, the cellular mechanism of NLRP3 inflammasome activation during *S. aureus* infection remains unknown. In the present study, we found that spleen tyrosine kinase (Syk) and c-Jun N-terminal kinase (JNK) were rapidly phosphorylated during *S. aureus* infection. Moreover, a Syk/JNK inhibitor and Syk/JNK siRNA not only reduced NLRP3 inflammasome-associated molecule expression at the protein and mRNA levels, apoptosis-associated speck-like protein containing a caspase-recruitment domain (ASC) speck formation, and interleukin-1β (IL-1β), and IL-18 release but also rescued the decreased NIMA-related kinase 7 (NEK7) expression level following suppression of the NEK7-NLRP3 interaction in macrophages. Interestingly, Syk/JNK phosphorylation levels and NLRP3 inflammasome-associated molecule expression were decreased by blockade of K^+^ efflux. Furthermore, activation of the NLRP3 inflammasome and a lower NEK7 protein level were found *in vivo* upon *S. aureus* infection. Taken together, our data indicated that *S. aureus* infection induces a K^+^ efflux/Syk/JNK/NEK7-NLRP3 signaling pathway and the subsequent activation of the NLRP3 inflammasome for the release of proinflammatory cytokines. This study expands our understanding of the basic molecular mechanism regulating inflammation and provides potential value for anti-infective drug development against *S. aureus* infection.

## Introduction

*Staphylococcus aureus* (*S. aureus*), a gram-positive foodborne pathogen, is carried by approximately 30% of the world population (1). As one of the most common nosocomial pathogens, *S. aureus* causes a variety of invasive infections (2), such as pneumonia, endocarditis, bacteremia, and sepsis (3), which can lead to death (4). Moreover, with the increasing rates of antibiotic resistance, morbidity and mortality are increasing (5). Therefore, studying the mechanism induced by *S. aureus* infection is extremely important for resisting *S. aureus* infection.

NLRP3 inflammasomes, which are multiprotein complexes, play a key role in the innate immune system against various pathogens. NLRP3 inflammasome activation is triggered by many stimuli, such as pathogen-associated molecular patterns (PAMPs), adenosine triphosphate (ATP), and toxins (6, 7), which lead to the aggregation of ASC and the proteolytic activation of pro-caspase-1 in macrophages, following IL-1β and IL-18 maturation and secretion (8, 9). In contrast, dietary selenium can attenuate *S. aureus*-induced mastitis by inhibiting the NLRP3 inflammasome (10). Despite these advances, the exact molecular mechanism of *S. aureus* infection-induced NLRP3 inflammasome activation remains uncertain.

NIMA-related kinase 7 (NEK7), which plays a crucial role in mitosis entry, cell cycle progression, cell division, and mitotic processes, has been proven to be a vital mediator during inflammasome activation in hepatocellular carcinoma (11). Moreover, potassium efflux is a common event in NLRP3 inflammasome activation (12), and NEK7-mediated assembly and activation of the NLRP3 inflammasome are downstream of potassium efflux in ventilator-induced lung injury (VILI) (13). However, whether *S. aureus* infection induces potassium efflux and the formation of the NEK7-NLRP3 complex needs to be fully elucidated.

In our previous studies, we found that Syk/JNK activated the NLRP3 inflammasome in diabetic cardiomyopathy (DCM) and diabetic nephropathy (DN) (14, 15). However, the role of Syk and JNK in infectious diseases remains unknown. Interestingly, a recent study found that sulforaphane reduces intracellular survival of *S. aureus* in macrophages through inhibition of JNK (16). Therefore, we hypothesized that Syk/JNK/NLRP3 plays an indispensable role upon *S. aureus* infection.

In the present study, we demonstrated that NEK7 and NLRP3 inflammasome activation was involved in *S. aureus* infection *in vivo*. Moreover, we are the first to provide evidence that Syk/JNK promotes NLRP3 inflammasome activation by regulating the formation of NEK7-NLRP3 in J774A.1 cells and peritoneal exudate cells (PECs), which was mediated by potassium efflux. In summary, the K^+^ efflux/Syk/JNK/NEK7-NLRP3 signaling pathway plays a critical role in defending against *S. aureus*-infection.

## Results

### NLRP3 Inflammasome Was Activated in *S. aureus*-Induced Mouse Pneumonia

**Figure 1:**
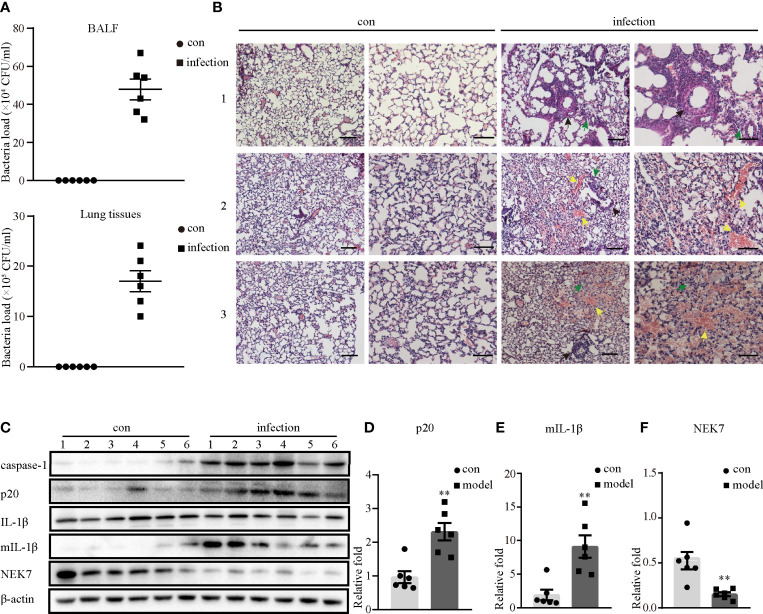
NLRP3 inflammasome was activated in *S. aureus*-induced mouse pneumonia. 6-8 weeks old female mice (C57BL/6) were randomly divided into the control group and the infection group, groups were both challenged with indicate stimulate groups. The infection group mice were challenged with 25 µl of a 10^9^ CFU/mL suspension of *S. aureus* by intranasal administration, while the mice in the control group were given an equal volume of sterile PBS solution. **(A)** Bacterial load of BALF or lungs were determined in the control group and the infection group by CFU assay. **(B)** Histological examination for lungs were visualized by H&E stain (×100 magnification, first column and third column; ×200 magnification, second column and forth column). The arrows represent different inflammatory pathological changes in the infection group. (Scale bar: 100 µm.) **(C)** Western blot showing the protein levels of caspase-1, p20, pro-IL-1β, mIL-1β and NEK7. **(D–F)** Densitometry analysis of p20, mIL-1β and NEK7. Control group *n*=6, infection group *n*=6. Data shown are means ± SEMs; **P < 0.01 *vs.* control group.

To test the mechanism by which *S. aureus* infection activates the NLRP3 inflammasome, we first established an *S. aureus* infectious pneumonia model and determined the colony-forming units (CFUs) of *S. aureus* in bronchoalveolar lavage fluid (BALF) and lung tissues. The average bacterial load in BALF was approximately 4.7×10^5^ CFU/ml and the average bacterial load in the lung was approximately 1.7×10^6^ CFU/ml, which demonstrated that the *S. aureus* infection pneumonia model had been built successfully (**Figure 1A**) (17). Additionally, H&E stain of the lungs in the infection group showed that the alveolar wall was thickened, the structure of the lung was severely damaged and lymphocytes were attached to the vessel wall (**Figure 1B**, black arrow), in addition, lymphocytes also accumulated outside of blood vessels and bronchi (**Figure 1B**, green arrow), and erythrocytes accumulated in the bronchi (**Figure 1B**, yellow arrow). However, these phenomena were not observed in the control group, suggesting that the lungs in the *S. aureus* infection group underwent a series of inflammatory pathological changes. Moreover, we quantify the pathological score in the lungs based on these pathological phenomena (**Table 1**).

**Table 1:** Pathological score in the lung.

Sample number	Alveolar congestion	Hemorrhage	Inflammatory cell infiltration	Alveolar wall thickness	Total score
1	0	0	0	0	0
2	0	0	0	0	0
3	0	0	0	0	0
4	0	1	3	1	5
5	2	2	2	1	7
6	3	3	1	0	7

In short, on a scale of 0 to 4 for four pathological parameters were scored: (1) alveolar congestion, (2) hemorrhage, (3) inflammatory cell infiltration, and (4) alveolar wall thickness. 0 points represent normal lung, and scores of 1, 2, 3, and 4 represent mild (<25%), moderate (25%-50%), severe (50%-75%), and very severe (>75%) lung involvement, respectively. The total score was based on the sum of all score. Control group (1-3); infection group (4-6).

Next, we investigated whether inflammatory pathological changes were correlated with the NLRP3 inflammasome, and determined the NLRP3 inflammasome-related protein expression levels in the lungs. As shown in **Figures 1C–F**, the protein expression levels of activated caspase-1 (p20) and mature IL-1β (mIL-1β) in the infection group were significantly higher than those in the control group. Moreover, NEK7 expression at the protein level was markedly decreased in the infection group. Taken together, these data demonstrated that the NLRP3 inflammasome was activated in *S. aureus*-induced pneumonia, accompanied by decreased NEK7 protein levels.

### NEK7 Was Vital in *S. aureus* Infection-Induced NLRP3 Inflammasome Activation

**Figure 2:**
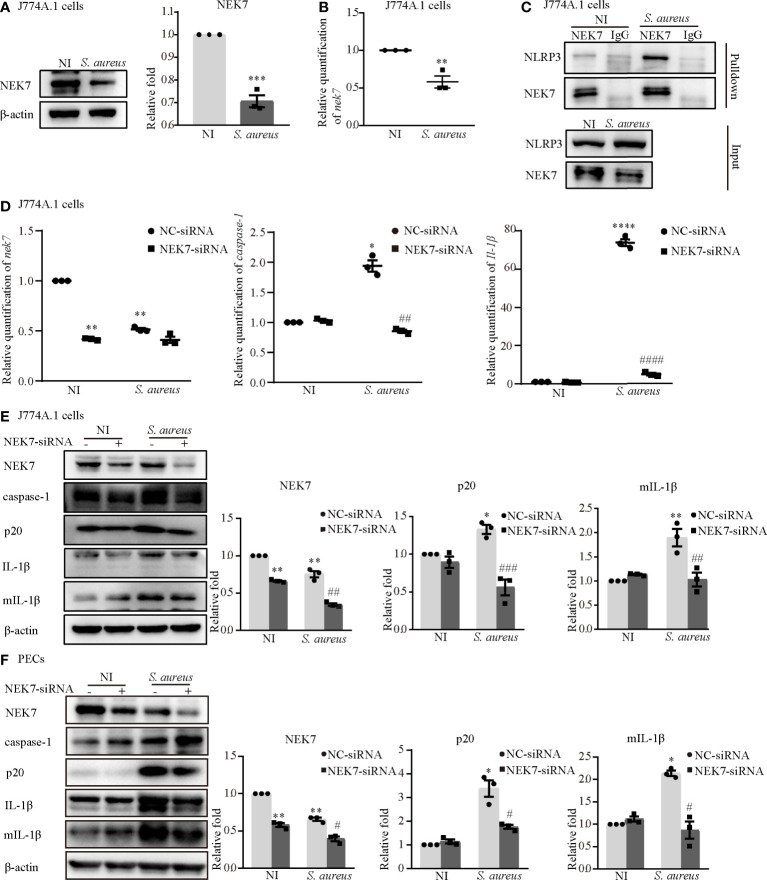
NEK7 was vital in *S. aureus* infection-induced NLRP3 inflammasome activation. The J774A.1 cells were infected with *S. aureus* (MOI of 1) for 24 h. **(A)** The protein expression level of NEK7 were detected by western blot and analyzed by relative densitometric quantification. **(B)** The mRNA expression level of *nek7* were detected by RT-PCR. **(C)** The NEK7-NLRP3 ineraction were detected by coimmunoprecipitation. **(D)** Cells were pretreated with or without NEK7-siRNA for 48 h. The mRNA expression levels of *caspase-1*, *Il-1β* and *nek7* were examined by RT-PCR. **(E, F)** The protein expression levels of caspase-1, p20, pro-IL-1β, mIL-1β and NEK7 were detected by western blot in J774A.1 cells **(E)** and PECs **(F)** and analyzed by relative densitometric quantification. Similar results were obtained in three independent experiments in data. Data shown are means ± SEMs; *P < 0.05, **P < 0.01, ***P < 0.001, ****P < 0.0001 *vs*. non-infection control group, ^#^ *P* < 0.05, ^##^ *P* < 0.01, ^###^ *P* < 0.001, ^####^ *P* < 0.0001 *vs*. infection control group.

To understand the role of NEK7 more deeply, we assessed the protein and mRNA expression levels of NEK7 in macrophages infected with *S. aureus*. The results showed that the protein and mRNA expression of NEK7 was reduced in the infection group of J774A.1 cells (**Figures 2A, B**). Notably, the NLRP3-NEK7 compound was obviously increased upon *S. aureus* infection, as detected by coimmunoprecipitation (**Figure 2C**). Moreover, NEK7-siRNA attenuated both the mRNA levels of *caspase-1* and *Il-1β* (**Figure 2D**) and the protein expression of activated caspase-1 (p20) and mature-IL-1β (mIL-1β) (**Figure 2E**) in J774A.1 cells infected with *S. aureus*. Similar results were observed in PECs (**Figure 2F**). These findings indicated that NEK7 was necessary for NLRP3 inflammasome activation.

### Syk and JNK Promoted NLRP3 Inflammasome Activation Upon *S. aureus* Infection

**Figure 3:**
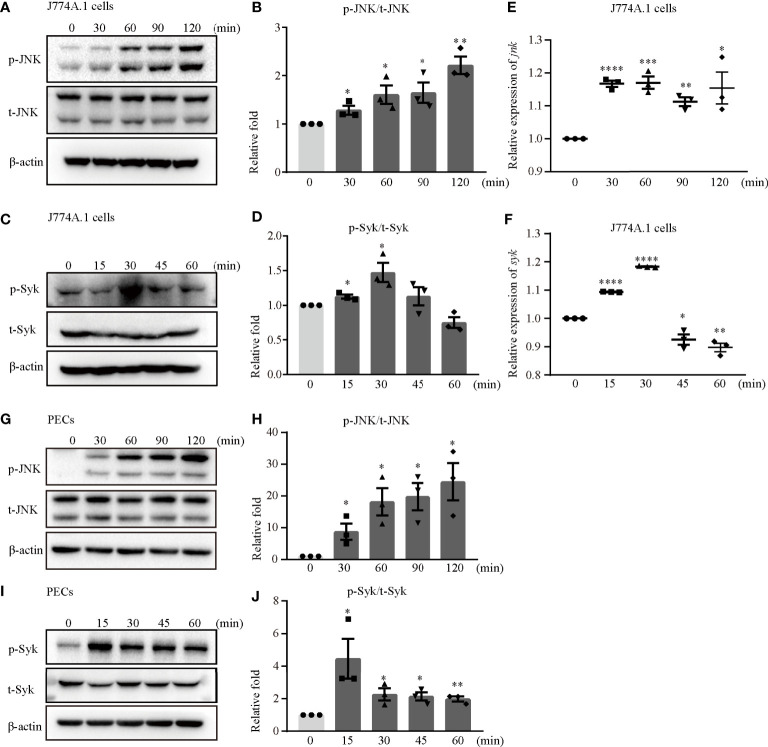
JNK and Syk were phosphorylated upon *S. aureus* infection. J774A.1 cells **(A–F)** and PECs **(G–J)** were infected with *S. aureus* at an MOI of 5 for different time. The cell lysates and total cellular RNA were collected. **(A–D)** Total and phosphorylation levels of JNK **(A)** and Syk **(C)** were detected by western blot. p-JNK/t-JNK **(B)** and p-Syk/t-Syk **(D)** were analyzed by relative densitometric quantification. **(E, F)** The mRNA expression levels of *jnk* **(E)** and *syk* **(F)** were quantified by RT-PCR. **(G–J)** The protein levels of t-JNK, p-JNK **(G)** and t-Syk, p-Syk **(I)** were detected by western blot. p-JNK/t-JNK **(H)** and p-Syk/t-Syk **(J)** were analyzed by relative densitometric quantification. Similar results were obtained in three independent experiments in data. Data shown are means ± SEMs; *P < 0.05, **P < 0.01, ***P < 0.001, ****P < 0.0001 *vs*. non-infection group.

The Syk/JNK/NLRP3 signaling pathway was involved in diabetic cardiomyopathy and diabetic nephropathy in our previous study (14, 15). Hence, we confirmed whether Syk and JNK mediated NLRP3 inflammasome activation during *S. aureus* infection. Interestingly, the phosphorylation levels of JNK and Syk were increased in a time-dependent manner in both J774A.1 cells (**Figures 3A–D**) and PECs (**Figures 3G–J**) during *S. aureus* infection. Moreover, the mRNA levels of *syk* and *jnk* were also increased starting at 15 minutes and 30 minutes after *S. aureus* infection (**Figures 3E, F**). These results indicated that Syk and JNK were involved in *S. aureus* infection.

**Figure 4:**
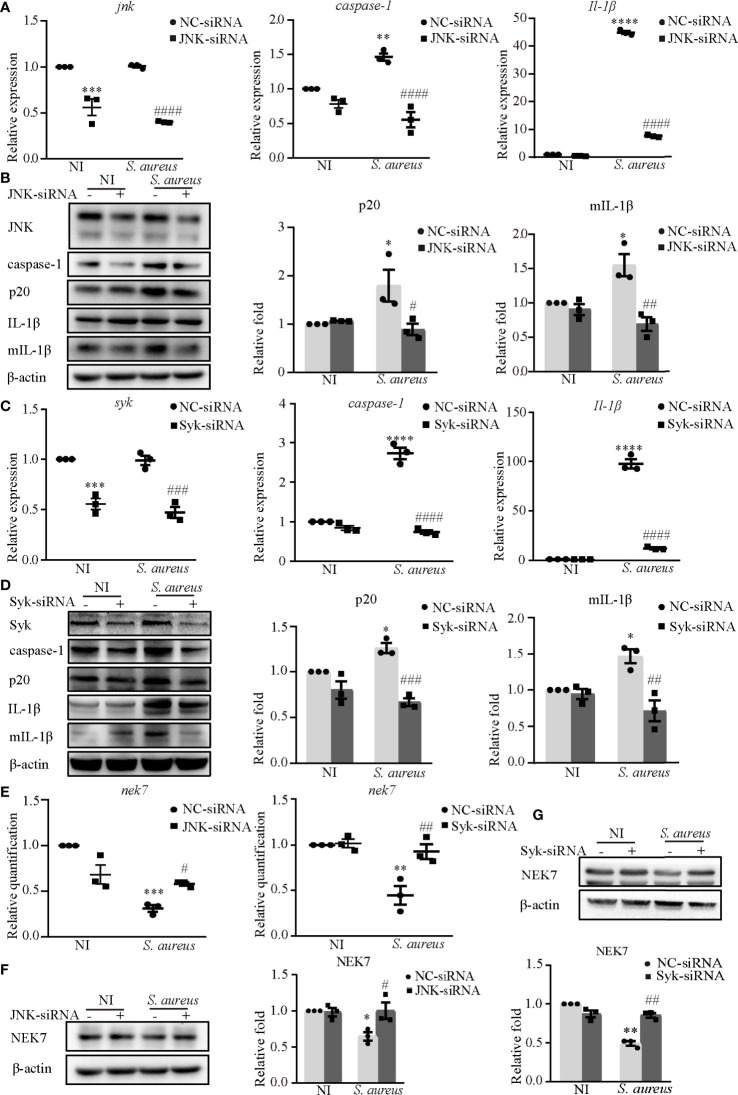
Syk and JNK promoted the NLRP3 inflammasome activation upon *S. aureus* infection. J774A.1 cells were infected with *S. aureus* (MOI of 1) for 24 h with or without JNK or Syk-siRNA pretreatment for 48 h. **(A)** The mRNA expression levels of *caspase-1*, *Il-1β* in the cells pretreated with or without JNK-siRNA were quantified by RT-PCR. **(B)** The protein expression levels of caspase-1, p20, pro-IL-1β and mIL-1β were detected by western blot and analyzed using densitometry. **(C)** The mRNA levels of *caspase-1* and *Il-1β* in the cells pretreated with or without Syk-siRNA were quantified by RT-PCR. **(D)** The protein expression levels of caspase-1, p20, pro-IL-1β and mIL-1β were detected by western blot and analyzed using densitometry. **(E)** The mRNA expression level of *nek7* was examined by RT-PCR with or without JNK/Syk-siRNA transfected. **(F, G)** The protein expression level of NEK7 was detected by western blot and analyzed using densitometry by transfected with or without JNK-siRNA **(F)** or Syk-siRNA **(G)**. Similar results were obtained in three independent experiments in data. Data shown are means ± SEMs; *P < 0.05, **P < 0.01, ***P < 0.001, ****P < 0.0001 *vs.* non-infection control group, ^#^ *P* < 0.05, ^##^ *P* < 0.01, ^###^ *P* < 0.001, ^####^ *P* < 0.0001 *vs*. infection control group.

Furthermore, JNK-siRNA or Syk-siRNA attenuated the *S. aureus* infection induced mRNA expression levels of *caspase-1* and *Il-1β* (**Figures 4A, C**). The protein expression of p20 and mIL-1β was significantly decreased in cells exposed to *S. aureus* following JNK-siRNA (**Figure 4B**) or Syk-siRNA (**Figure 4D**) treatment. These data strongly indicated that JNK and Syk played a key role in NLRP3 inflammasome activation.

In our present study, NEK7 was involved in NLRP3 inflammasome activation upon *S. aureus* infection. Hence, we hypothesize that Syk and JNK may be linked to NEK7. Interestingly, the decreased mRNA expression level (**Figure 4E**) and protein expression level of NEK7 (**Figures 4F, G**) were rescued by JNK-siRNA or Syk-siRNA during *S. aureus* infection, indicating that NEK7 was downregulated by JNK and Syk in NLRP3 inflammasome activation.

### Phosphorylated JNK Activated the NLRP3 Inflammasome by Accelerating NEK7-NLRP3 Compound Formation Upon *S. aureus* Infection

**Figure 5:**
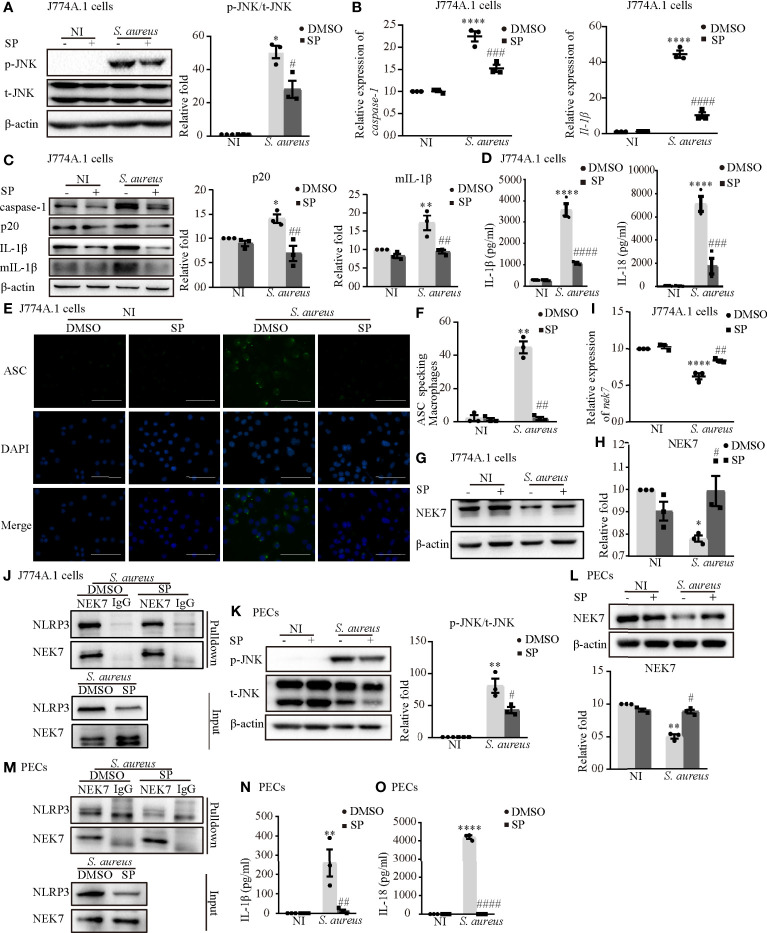
Phosphorylated JNK activated the NLRP3 inflammasome by accelarating NEK7-NLRP3 compound formation upon *S. aureus* infection. J774A.1 cells **(A–J)** and PECs **(K–O)** pretreated with or without JNK inhibitor (SP 600125) at the final concentration of 20 μmol/L for 2 h followed by *S. aureus* infecton (MOI of 1 or 5) for 24 h or 2 h. **(A)** The protein levels of t-JNK and p-JNK were detected by western blot and analyzed by relative densitometric quantification in J774A.1 cells and PECs **(K)**. **(B)** The mRNA expression levels of *caspase-1*, *Il-1β* were quantified by RT-PCR. **(C)** The protein expression levels of caspase-1, p20, pro-IL-1β and mIL-1β were examined by western blot and analyzed by relative densitometric quantification. **(D)** The supernatants were collected at 48 h after *S. aureus* infection. The levels of IL-1β and IL-18 in supernatants were detected by ELISA in J774A.1 cells and PECs **(N, O)**. **(E)** Confocal immunofluorescence imaged endogenous ASC specks. Cells were fixed and stained for ASC (Green), DAPI (Blue). (Scale bar: 100 µm.) **(F)** The percentage of J774A.1 cells with ASC specks were quantificated. More than 200 cells from at least 5 different fields were counted for ASC speck formation. **(G, H)** The protein expression level of NEK7 was examined by western blot and analyzed by relative densitometric quantification in J774A.1 cells and PECs **(L)**. **(I)** The mRNA expression level of *nek7* was examined by RT-PCR. **(J)** Coimmunoprecipitation analysis of NLRP3 interaction with NEK7 in J774.A cells and PECs **(M)**. Similar results were obtained in three independent experiments in data. Data shown are means ± SEMs; *P < 0.05, **P < 0.01, ****P < 0.0001 *vs.* non-infection control group, ^#^ *P* < 0.05, ^##^ *P* < 0.01, ^###^ *P* < 0.001, ^####^ *P*< 0.0001 *vs*. infection control group.

To further confirm the role of p-JNK in NLRP3 inflammasome activation, both J774A.1 cells and PECs were pretreated with the JNK inhibitor (SP) or a DMSO control. SP significantly reduced the phosphorylation degree of JNK in both J774A.1 cells (**Figure 5A**) and PECs (**Figure 5K**). The mRNA levels of *caspase-1* and *Il-1β* were decreased following SP pretreatment during *S. aureus* infection (**Figure 5B** and **Figure S1A**). Moreover, SP also attenuated the *S. aureus*-induced increase of p20 and mIL-1β in J774A.1 cells and PECs (**Figure 5C** and **Figure S1B**). In addition, the proinflammatory cytokines IL-1β and IL-18 in the supernatants of J774A.1 cells (**Figure 5D**) and PECs (**Figures 5N, O**) were substantially increased compared with those in the noninfected control group, whereas SP reduced the concentrations of IL-1β and IL-18 in the supernatant.

Upon NLRP3 inflammasome activation, ASC is recruited to the cytosol where it self-aggregates and forms specks, subsequently leading to caspase-1 activation (18). In **Figures 5E, F** and **Figure S1C**, we found that the number of ASC speck-positive cells in the infection group was much greater than that in the noninfection group through fluorescence microscopy. Inhibition of JNK phosphorylation by SP resulted in a significant reduction in *S. aureus* induced ASC speck formation in J774A.1 cells and PECs. Taken together, these results suggested that *S. aureus*-induced activation of the NLRP3 inflammasome and secretion of the proinflammatory cytokines IL-1β and IL-18 were dependent on p-JNK.

Whether p-JNK regulates NLRP3 inflammasome activation *via* NEK7 is poorly understood. In J774A.1 cells, the protein and mRNA expression of NEK7 was upregulated in the presence of SP during *S. aureus* infection (**Figures 5G–I**). Furthermore, the *S. aureus* induced NLRP3-NEK7 compound was obviously decreased by SP pretreatment (**Figure 5J**). Consistently, SP greatly increased the protein level of NEK7 and reduced NLRP3-NEK7 expression in PECs (**Figures 5L, M**). These results indicated that p-JNK activated the NLRP3 inflammasome by accelerating NEK7-NLRP3 compound formation upon *S. aureus* infection.

### Phosphorylated Syk Was Involved in *S. aureus* Infection Induced NEK7-NLRP3 Signaling Pathway Activation

**Figure 6:**
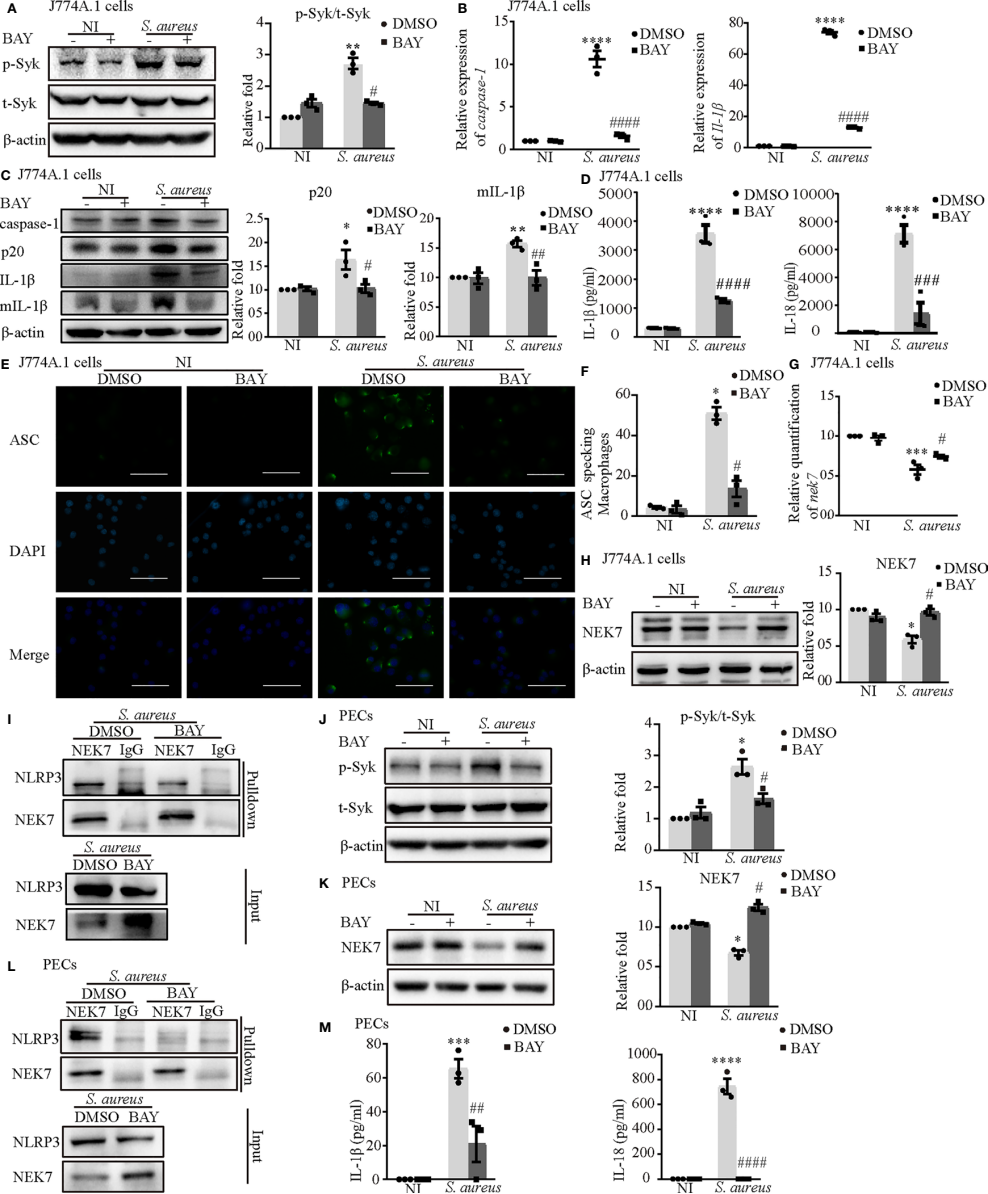
Phosphorylated Syk was involved in *S. aureus* infection-induced NEK7-NLRP3 signaling pathway activation. J774A.1 cells **(A–I)** and PECs **(J–M)** pretreated with or without Syk inhibitor (BAY 613606) at the final concentration of 1 μmol/L for 2 h followed by *S. aureus* infecton (MOI of 1 or 5) for different time. **(A)** The protein levels of t-Syk and p-Syk were detected by western blot and analyzed by relative densitometric quantification followed by *S. aureus* infection (MOI of 5) for 30 min in J774A.1 cells and PECs **(J)**. **(B)** The mRNA expression levels of *caspase-1* and *Il-1β* were quantified by RT-PCR. **(C)** The protein expression levels of caspase-1, p20, pro-IL-1β and mIL-1β were detected by western blot and analyzed by relative densitometric quantification. **(D)** The supernatants were collected at 48 h after *S aureus* infection. IL-1β and IL-18 in the supernatants were determined by ELISA in J774A.1 cells and PECs **(M)**. **(E)** The ASC specks formation were imaged *via* fluorescence microscopy. (Scale bar: 100 µm.). **(F)** ASC specks were quantitied in more than 200 cells from at least 5 different fields. **(G)** The mRNA expression level of *nek7* was examined by RT-PCR. **(H)** The protein expression level of NEK7 was detected by western blot and analyzed by relative densitometric quantification in J774A.1 cells and PECs **(K)**. **(I)** The NEK7-NLRP3 compound were analysed by coimmunoprecipitation in J774A.1 cells and PECs **(L)**. Similar results were obtained in three independent experiments in data. Data shown are means ± SEMs; *P < 0.05, **P < 0.01, ***P < 0.001, ****P < 0.0001 *vs*. non-infection control group, ^#^ *P* < 0.05, ^##^ *P* < 0.01, ^###^ *P* < 0.001, ^####^ *P* < 0.0001 *vs.* infection control group.

First, we confirmed that the inhibitor of Syk (BAY) reduced Syk phosphorylation in both J774A.1 cells (**Figure 6A**) and PECs (**Figure 6J**). The increased mRNA expression of *caspase-1* and *Il-1β* induced by *S. aureus* infection in J774A.1 cells and PECs was significantly reduced following pretreatment with an inhibitor of Syk (BAY) (**Figure 6B** and **Figure S1D**). Moreover, the protein expression of p20 and mIL-1β showed similar tendencies (**Figure 6C** and **Figure S1E**). Furthermore, the secretion of IL-1β and IL-18 from J774A.1 cells (**Figure 6D**) and PECs (**Figure 6M**) in the *S. aureus* infection group was significantly increased compared with that in the noninfection group, while in comparison, the expression level of IL-1β and IL-18 in BAY pretreatment infection group was significantly decreased. In addition, BAY also reduced the formation of ASC specks in J774A.1 cells and PECs upon *S. aureus* infection (**Figures 6E, F** and **Figure S1F**). Taken together, these results suggested that the *S. aureus*-induced activation of the NLRP3 inflammasome and secretion of proinflammatory cytokines were dependent on p-Syk.

Consistent with our hypothesis, BAY obviously upregulated the mRNA and protein levels of NEK7 (**Figures 6G, H**) and decreased the NLRP3-NEK7 complex in J774A.1 cells (**Figure 6I**) during *S. aureus* infection. Likewise, BAY also greatly increased the protein level of NEK7 and reduced NLRP3-NEK7 compound in PECs (**Figures 6K, L**). These results confirmed that p-Syk was involved in the NEK7-NLRP3 signaling pathway during *S. aureus* infection.

### Syk/JNK Signaling Was Critical for Activation of the NLRP3 Inflammasome Upon *S. aureus* Infection

Notably, the phosphorylation time of Syk was earlier than that of JNK and the expression of p-Syk gradually decreased when JNK was phosphorylated (**Figure 3**), so we hypothesized that the process of JNK phosphorylation may consume the amount of p-Syk.

**Figure 7:**
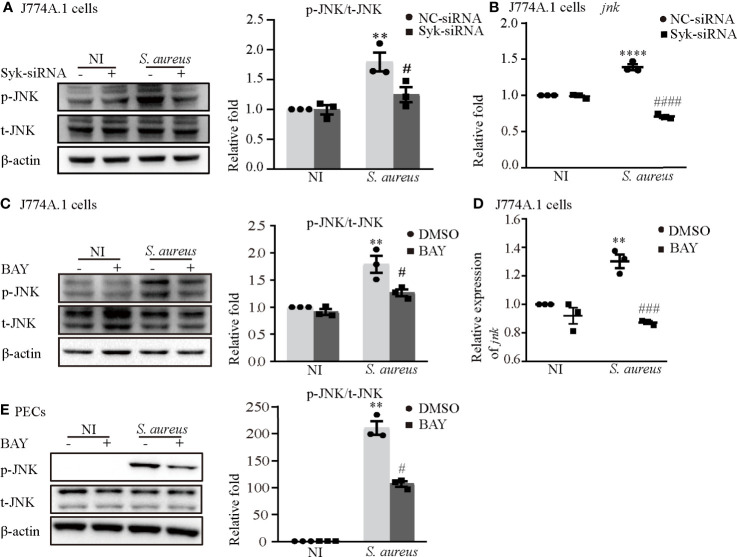
Syk/JNK signaling was critical for activation of the NLRP3 inflammasome upon *S. aureus* infection. Macrophages were infected with *S. aureus* (MOI of 5) for 2 h by pretreated with or without Syk-siRNA **(A**, **B)** for 48 h or Syk inhibitor (BAY) **(C–E)** for 2h. **(A)** The protein expression level of p-JNK was detected by western blot and analyzed by relative densitometric quantification in J774A.1 cells. **(B)** The mRNA expression level of *jnk* was quantified by RT-PCR. **(C)**The protein expression level of p-JNK was detected by western blot and analyzed by relative densitometric quantification in J774A.1 cells and PECs **(E)**. **(D)** The mRNA expression level of *jnk* was quantified by RT-PCR. Similar results were obtained in three independent experiments in data. Data shown are means ± SEMs; **P < 0.01, ****P < 0.0001 *vs*. non-infection control group, ^#^ *P* < 0.05, ^###^ *P* < 0.001, ^####^ *P* < 0.0001 *vs.* infection control group.

To clarify the relationship between JNK and Syk during *S. aureus* infection, we assessed the expression level of JNK with or without Syk-siRNA or Syk inhibitor pretreatment in J774A.1 cells or PECs. As shown in **Figures 7A, B**, the phosphorylation level of JNK and the mRNA expression of *jnk* induced by *S. aureus* infection were obviously reduced in J774A.1 cells pretreated with Syk-siRNA. Moreover, the Syk inhibitor also reduced phosphorylation level of JNK in J774A.1 cells and PECs (**Figures 7C, E**) and the mRNA expression of *jnk* (**Figure 7D**) upon *S. aureus* infection. Combined with the results of **Figure 3**, it was clear that Syk was an important regulator of JNK phosphorylation during *S. aureus* infection.

Syk/JNK signaling was critical for activation of the NLRP3 inflammasome by regulating NEK7-NLRP3 compound formation upon *S. aureus* infection

### Blockade of Potassium Efflux Decreased the Phosphorylation Levels of Syk and JNK Upon *S. aureus* Infection

**Figure 8:**
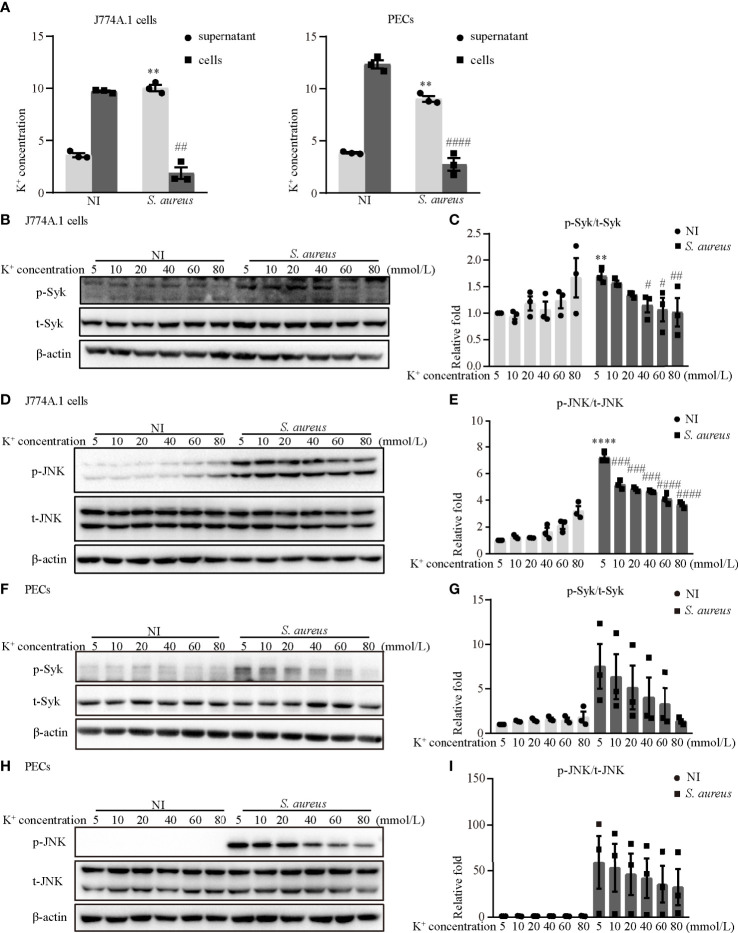
Blockade of potassium efflux decreased the phosphorylation levels of Syk and JNK upon *S. aureus* infection. **(A)** K^+^ concentration in the supernatant and cells of J774A.1 cells and PECs infected with *S. aureus* (MOI of 1) for 24 h **(B–I)** J774A.1 cells and PECs were infected with *S. aureus* (MOI of 5) for different time in the presence of following treating with increasing concentration of KCl (5 mM-80 mM). The cell lysates were collected at 0.5 h and 2 h. **(B)** The protein levels of t-Syk and p-Syk were detected by western blot in J774A.1 cells and PECs **(F)**. **(C)** Relative densitometric quantification analysis of p-Syk/t-Syk in J774A.1 cells and PECs **(G)**. **(D)** The protein levels of t-JNK and p-JNK were detected by western blot in J774A.1 cells and PECs **(H)**. **(E)** Relative densitometric quantification analysis of p-JNK/t-JNK in J774A.1 cells and PECs **(I)**. Similar results were obtained in three independent experiments in data. Data shown are means ± SEMs; **P < 0.01, ****P < 0.0001 *vs*. non-infection 5 mM group, ^#^ *P* < 0.05, ^##^ *P* < 0.01, ^###^ *P* < 0.001, ^####^ *P* < 0.0001 *vs.* infection 5 mM group.

It has been generally accepted that K^+^ efflux triggers NLRP3 activation in response to some stimuli (12). To assess whether activation of the NLRP3 inflammasome was dependent on K^+^ efflux and to explore the molecular mechanism during *S. aureus* infection, J774A.1 cells and PECs were pretreated with increasing concentrations of potassium chloride (KCl). First, the results showed that the K^+^ concentration in the supernatant in the infection group was much higher than that in the noninfection group, whereas the K^+^ concentration in cells in the infection group was much lower in both J774A.1 cells and PECs (**Figure 8A**). Moreover, a high extracellular concentration of KCl inhibited the *S. aureus*-induced phosphorylation of Syk and JNK in a dose-dependent manner in both J774A.1 cells (**Figures 8B–E**) and PECs (**Figures 8F–I**). Notably, the phosphorylation levels of Syk and JNK were decreased in the 40 mM group and 10 mM group, respectively. These results suggested that K^+^ efflux might activate the NLRP3 inflammasome through phosphorylation of Syk and JNK.

### Blockade of Potassium Efflux Reduced NLRP3 Inflammasome Activation and NEK7 Expression

**Figure 9:**
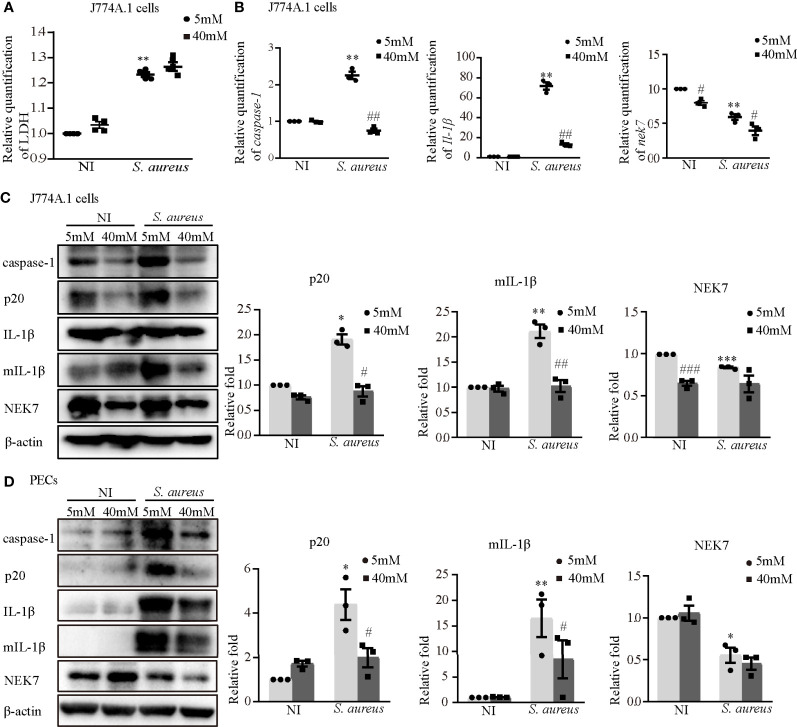
Blockade of potassium efflux downregulated NLRP3 inflammasome activation and NEK7 expression. J774A.1 cells **(A–C)** and PECs **(D)** were infected with *S. aureus* infection (MOI of 1) for 24 h in the presence of different concentration of KCl (5 mM or 40 mM). **(A)** The level of LDH in the supernatants were detected. **(B)** The mRNA expression levels of *caspase-1*, *Il-1β* and *nek7* were examined by RT-PCR. **(C)** The protein expression levels of caspase-1, p20, pro-IL-1β, mIL-1β and NEK7 were detected by western blot and analyzed by relative densitometric quantification in J774A.1 cells and PECs **(D)**. Similar results were obtained in three independent experiments in data. Data shown are means ± SEMs; *P < 0.05, **P < 0.01, ***P < 0.001 vs. non-infection control group, ^#^ *P* <0.05, ^##^ *P* < 0.01, ^###^ *P* < 0.001 *vs*. infection control group.

To further determine the role of K^+^ efflux in NLRP3 inflammasome activation upon *S. aureus* infection, J774A.1 cells and PECs were cultured in medium with or without 40 mM KCl for 24 h. There was no significant difference in cell viability between the 5 mM group and the 40 mM group in the J774A.1 cells, while the cell viability was decreased upon *S. aureus* infection (**Figure 9A**). Compared to the 5 mM group, the mRNA expression levels of *caspase-1*, *Il-1β* and *nek7* in the 40 mM group were obviously decreased in J774A.1 cells upon *S. aureus* infection (**Figure 9B**). Furthermore, 40 mM KCl also significantly reduced the protein expression level of p20, mIL-1β and NEK7 in both J774A.1 cells (**Figure 9C**) and PECs (**Figure 9D**). In summary, these results indicated that the K^+^ efflux/Syk/JNK signaling pathway was involved in NLRP3 inflammasome activation by regulating NLRP3-NEK7 complex formation during *S. aureus* infection.

## Discussion

As an opportunistic pathogen, *S. aureus* infection is a main cause of invasive infections, which can range from superficial skin infections to potentially life-threatening systemic diseases. A series of inflammatory processes can be observed during *S. aureus* infection, such as cytokine release, tissue fibrosis, and neutrophil aggregation (19). Herein we showed that many inflammatory pathological phenomena appeared in the lung tissue of mice in the *S. aureus* infection group following NLRP3 inflammasome-associated molecules activation (**Figure 1** and **Table 1**), suggesting that inflammation may be induced by NLRP3 inflammasome activation. Thus, the exact mechanism of NLRP3 inflammasome activation needs to be studied more deeply at the cellular level.

To gain a better understanding of the molecular mechanism of NLRP3 inflammasome activation, we focused on Syk and JNK because they particapate in NLRP3 inflammasome activation in DCM and DN (14, 15). In addition, a recent report found that the Dectin-2/Syk/JNK/NF-κB pathway induces NLRP3 and pro-IL-1β expression in Kawasaki disease (20), which is similar to our conclusion. Furthermore, Syk is known to be critical for IL-1β released during *Toxoplasma gondii* infection (21) and important in TLR4-mediated signaling upon LPS stimulation. Herein, we demonstrated that Syk and JNK were phosphorylated in *S. aureus*-infected macrophages (**Figure 3**), and that genetic knockdown of Syk or JNK in J774A.1 cells not only significantly reduced the transcripts of caspase-1 and IL-1β but also decreased the protein expression of p20 and mIL-1β (**Figures 4A–D**). In addition, similar results were obtained using phosphorylation inhibitors of Syk or JNK (**Figures 5B, C**; **Figures 6B, C** and **Figures S1A, B, D, E**). Moreover, our data showed that p-Syk/p-JNK promoted the formation of ASC specks and mediated caspase-1 activation, IL-1β maturation and secretion (**Figures 5D–F, N, O**; **6D–F, M** and **Figures S1C, F**), indicating that the phosphorylation levels of Syk and JNK play a key role in NLRP3 inflammation activation upon *S. aureus* infection. Similarly, a previous study revealed that Syk enhances ASC oligomerization and regulates NLRP3 inflammasome activation *via* ASC phosphorylation at the Y146 and Y187 residues (22), although we have not confirmed the specific activation domain of ASC during *S. aureus* infection. Moreover, we demonstrated that Syk participated in this process by regulating JNK phosphorylation (**Figure 7**), indicating that Syk/JNK signaling is critical for activation of the NLRP3 inflammasome upon *S. aureus* infection.

NEK7, a serine/threonine kinase implicated in mitosis, shows lower protein and mRNA levels in systemic lupus erythematosus (SLE) patients (23). NEK7 is also necessary for NLRP3 inflammasome assembly and activation in bone marrow cells treated with LPS, followed by nigericin or ATP stimulation (24). Interestingly, our study found that the protein level of NEK7 was decreased in *S*. *aureus* induced mouse pneumonia (**Figures 1C, F**). However, whether NEK7 participates in *S. aureus* infection-induced NLRP3 inflammasome activation remains unknown.

In agreement with the *in vivo* results, our *in vitro* data showed that the expression of NEK7 in both types of macrophages was decreased during *S. aureus* infection, simultaneously, the formation of NEK7 and NLRP3 complexes was increased (**Figures 2A–C**). Consistently, some studies reported that the expression of NEK7 was decreased with the enhanced interaction of NEK7-NLRP3 under the stimulation of LPS and ATP (25), LPS and cytotoxic necrosis factor (CNF1) (26) or LPS and IgA ICs (27). Furthermore, *S. aureus* infection-induced expression of NLRP3 inflammasome-associated molecules were significantly decreased by NEK7-siRNA pretreatment (**Figures 2D–F**), suggesting that NEK7 participated in NLRP3 inflammasome activation. In addition, a previous study reported that NEK7 may be a switch between mitosis and the NLRP3 inflammasome, indicating that the interaction between NEK7 and NLRP3 is restricted to interphase of the cell cycle and that cells with activated NLRP3 inflammasomes cannot initiate mitosis (24). Based on above all, we speculated that the decreased expression of NEK7 may be due to the cell damage. As shown in **Figure 9A**, the LDH level in the culture supernatant was effectively increased. Unfortunately, we did not assess the growth cycle of the cells in our study.

To study the role of Syk/JNK in regulating NEK7-NLRP3 compound formation more deeply, we assessed the expression level of NEK7 with JNK/Syk-siRNA or JNK/Syk inhibitor pretreatment. Notably, our study demonstrated, for the first time, that the decreased mRNA and protein expression levels of NEK7 were rescued by JNK or Syk-siRNA (**Figures 4E–G**) and JNK or Syk inhibitors (**Figures 5G–I, L**; **Figures 6G, H, K**), indicating a role for Syk/JNK in regulating NEK7 expression in *S. aureus*-infected cells. At the same time, the inhibition of JNK or Syk in J774A.1 cells and PECs significantly reduced the interaction of NEK7-NLRP3 in *S. aureus* infection (**Figures 5J, M**; **Figures 6I, L**), suggesting that p-Syk/p-JNK accelerates NEK7-NLRP3 complex formation by downregulating NEK7 expression. In contrast, our recent study in *Listeria monocytogenes* infection found that the protein expression of NEK7 was increased (28). Taken together our findings in the present research suggest that NEK7 might play different roles in the infection of intracellular bacteria and extracellular bacteria, but we also need to pay attention to the cell cycle during infection.

To date, there are four main modes involved in activating NLRP3 inflammasome. One of them is the ion flux model. In this model, K^+^ efflux is widely accepted as an essential second step in the activation of NLRP3 inflammasomes, and this process is mediated by NEK7 during LPS and ATP costimulation (29, 30). Our data showed that K^+^ efflux increased upon *S. aureus* infection (**Figure 8A**), in addition, blockade of K^+^ efflux decreased the phosphorylation levels of Syk and JNK in both J774A.1 cells and PECs (**Figures 8B–I**). Moreover, the protein and mRNA level of NLRP3 inflammasome-associated molecules were all decreased when K^+^ efflux was inhibited (**Figure 9**), further suggesting that the NLRP3 inflammasome activation regulated by the Syk/JNK/NEK7 pathway was downstream of K^+^ efflux. Another mode of NLRP3 inflammasome activation is the reactive oxygen species (ROS) activation model. Mitochondrial ROSs are mediators of multiple activators of the NLRP3 inflammasome (31, 32), and the interaction between NEK7 and NLRP3 by ROS is K^+^ efflux-independent (21). However, macrophages are unable to effectively kill *S. aureus* internalized in mitochondria (33). Furthermore, cell lysosomal destruction and metabolites can also activate the NLRP3 inflammasome (34, 35). However, we did not consider the mechanism of *S. aureus* infection induced NLRP3 inflammasome activation from these aspects in the present study, and further studies are needed to verify this view.

NLRP3 inflammasome activation triggers caspase-1-dependent pyroptosis, which is a lytic form of programmed cell death that leads to the release of inflammatory cytokines (36), but there are very few studies about pyroptosis induced by *S. aureus* infection. Taken together with our present study, whether infection triggers pyroptosis to defend against bacteria needs further study. Furthermore, excessive inflammation may cause serious damage in normal tissue, so the anti-inflammatory mechanism needs to be activated to prevent it (37). Our previous study found that resveratrol (RSV) inhibits tumor progression by downregulating of NLRP3 (38), but whether RSV plays an anti-inflammatory role in *S. aureus* pneumonia to protect normal lung tissues needs further investigation.

Although our study of *S. aureus* infection induced NLRP3 inflammasome activation is more detailed, there are still some questions that need to be answered. It is well established that *S. aureus* employs a wide array of virulence factors, including surface proteins, pore-forming toxins, and alpha toxin (39). Moreover, alpha toxin potentiates opportunistic bacterial lung infections (40). Therefore, whether alpha toxin plays a key role in this process needs to be addressed in future studies. Furthermore, with the increasing mortality induced by antibiotic-resistant bacteria, such as methicillin-resistant *Staphylococcus aureus (MRSA)*, whose mortality is similar to that of acquired immune deficiency syndrome (AIDS) (41), whether the K^+^ efflux/Syk/JNK/NEK7-NLRP3 signaling pathway can be activated to defend against *MRSA* infection remains unknown.

**Figure 10:**
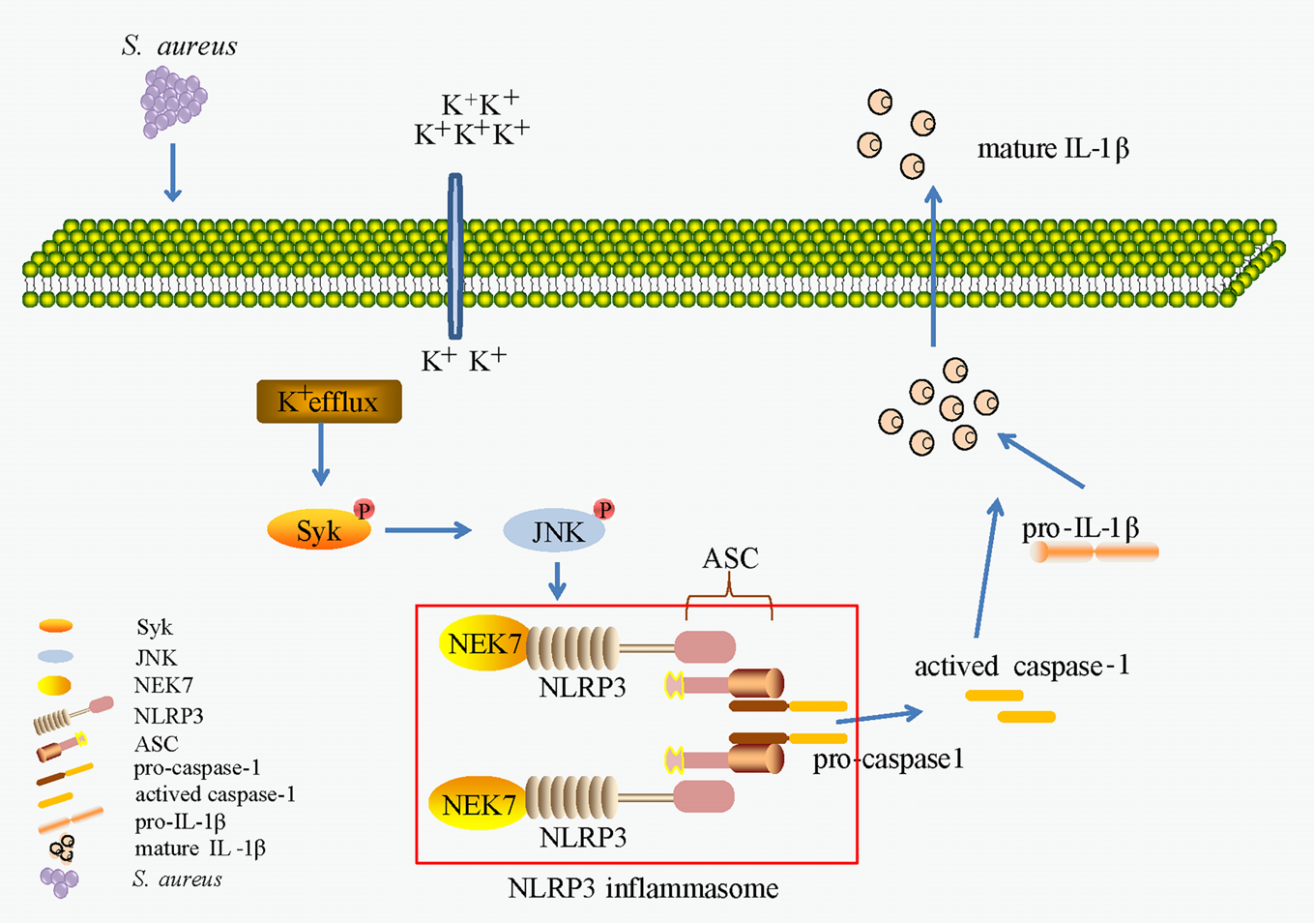
K^+^ efflux/Syk/JNK was important in NEK7-NLRP3 interaction and NLRP3 inflammasome activtion upon *S. aureus* infection.

In conclusion, as shown in **Figure 10**, our work provides evidence of a novel signaling pathway. Phosphorylation of Syk and JNK was dependent on potassium efflux during *S. aureus* infection, thereafter promoting NEK7-NLRP3 interaction and NLRP3 inflammasome activation through downregulation of NEK7, leading to the cleavage of pro-caspase-1 and maturation of IL-1β, and finally contributing to *S. aureus* induced-pneumonia. Our study provides a new dimension to understand the inflammation induced by *S. aureus* infection and may help to find potential therapeutic targets for drugs against *S. aureus* infection. However, future work should include more *in vivo* experiments to verify this conclusion.

## Materials and Methods

### Reagents and Antibodies

The JNK inhibitor II SP600125 (SP) was purchased from Merck KGaA, Inc. Syk inhibitor IV BAY 61-3606 was purchased from Santa Cruz Biotechnology, Inc. TRIzol^®^ reagent was purchased from Invitrogen Thermo Fisher Scientific, Inc. The H&E stain kit (cat. no. G1120), RIPA buffer (cat. no. R0020), DAPI solution (cat. no. C0060) and 4% paraformaldehyde (cat. no. P1110) were purchased from Beijing Solarbio Science & Technology Co., Ltd. (Beijing, China). Antibodies for phospho-JNK (cat. no. 9255s, 1:1,000), JNK (cat. no. 9252, 1:1,000), p-Syk (cat. no. 2710, 1:1,000), Syk (cat. no. 2712, 1:1,000), NLRP3 (cat. no. 15101, 1:1,000), rabbit IgG (cat. no. 2729) and caspase-1 (cat. no. 4199, 1:1,000) were purchased from Cell Signaling Technology, Inc. Antibodies for IL-1β (cat. no. ab9722, 1:2,000) and NEK7 (cat. no. ab133514, 1:2,000) were purchased from Abcam (Cambridge, UK). The antibody for β-actin (cat. no. sc-47778, 1:1,000) was purchased from Santa Cruz Biotechnology, Inc. An anti-ASC antibody (cat. no. A1170, 1:500) was obtained from Abclonal (Wuhan, China). HRP-conjugated anti-mouse immunoglobulin G (IgG) (cat. no. W4021, 1:5,000) and HRP-conjugated anti-rabbit IgG (cat. no. W4011, 1:5,000) were from Promega (Madison, WI, USA). Antibodies for FITC goat anti-rabbit IgG (cat. no. GR200G-02C, 1:100) were obtained from Tianjin Sungene Biotech Co., Ltd (Tianjin, China). The primers for *syk* (cat# MQP030294), *jnk* (cat# MQP099059), *caspase1*(cat# MQP028800), *Il-1β* (cat# MQP092978), *nek7* (cat# MQP101958) *and gapdh* (cat# MQP027158) were purchased from GeneCopoeia, Inc. (Beijing, China).

### Animals Care and *S. aureus* Infectious Mice Model

C57BL/6 WT female mice (age, 6-8 weeks old) were purchased from The Laboratory Animal Center of the Academy of Military Medical Sciences (Beijing, China, SYXK (Jin), 2019-0004). The mice were maintained under specific pathogen-free conditions of temperature (23 ± 5°C) and humidity (60 ± 5%) with alternating 12 h light/dark cycles. Clean drinking water and food were available freely. Mice were randomly divided into an infection group and a control group, with 6 mice per group. Infection group mice were challenged with 25 µl of a 10^9^ CFU/mL suspension of *S. aureus* (ATCC 25923) by intranasal administration, while the mice in the control group were given an equal volume of sterile PBS solution. After 24 hours, the number of viable *S. aureus* in both lung tissue and bronchoalveolar lavage fluid (BVLF) was counted by CFU counting assay. In addition, part of the lung tissue was homogenized for western blot and H&E stain.

### Cell Culture

The J774A.1 cell line (cat. no. ZQ0565) was purchased from Zhong Qiao Xin Zhou Biotechnology Co., Ltd. (Shanghai, China). J774A.1 cells were maintained in Dulbecco’s modified Eagle’s medium (DMEM) (Gibco, USA.) supplemented with 15% FBS (FBS, LONSERA, cat. no. s711-001s, Shanghai, China) and 1% streptomycin/penicillin. Cells were seeded at various densities in 6/12/96−well plates. After 2.5 h, the adherent cells were pretreated with or without Syk inhibitor (1 μM) or JNK inhibitor (20 μM) for 2 h, or siRNA targeting Syk, JNK or NEK7 (50 nM) for 48 h or KCl (5-80 mM), after which they were subsequently exposed to a noninfectious environment or an *S. aureus* infectious environment (MOI 1/MOI 5) for different times, followed by harvesting of supernatant and cell lysates for analysis.

### Isolation and Culture of Murine Peritoneal Exudate Cells

Mice were given a single intraperitoneal injection of 3 ml of 4% thioglycollate medium (Eiken Chemical, Tokyo, Japan). Three and a half days later, peritoneal exudate cells were collected and washed with RPMI 1640 medium (Gibco, USA). Cells were seeded in cell culture plates with RPMI 1640 medium containing 10% fetal bovine serum at 37°C and 5% CO_2_. After 2.5 h, adherent macrophages were used for experiments.

### Bacteria

*Staphylococcus aureus* (ATCC 25923) was grown overnight in brain heart infusion broth (BHI, Eiken Chemical Co., Ltd., Tokyo, Japan) at 37°C with shaking. One volume of the overnight culture (2 ml) was added to 100 volumes of fresh BHI broth and cultured for 4 to 5 h. The bacteria were washed, suspended in PBS containing 10% glycerol and stored in a -80°C refrigerator. Three and a half days later, we selected three tubes of *S. aureus* randomly, determined the concentration of bacteria by plating a 10-fold serial dilution of bacterial suspension on tryptic soy agar (TSA) plates and counted the colonies at 24 h after incubation at 37°C. For infections of the cultured cells, an MOI of 1 or 5 was used. After 1 h, gentamicin was added to the culture at a final concentration of 10 mg/L.

### Transient Transfection

J774A.1 cells were transfected with Syk, JNK or NEK7-siRNA (50 nM) using Lipofectamine™ 3000 (Thermo Fisher Scientific, Inc.) according to the manufacturer’s protocol to knockdown *syk*, *jnk*, and *nek7*, respectively; a scrambled siRNA, the negative control (NC)-siRNA, was used as a control in the experiment. The sequences for mouse Syk-siRNA were 5’-CCAGGUGGAAUAAUCUCAATT-3’ (sense) and 5’-UUGAGAUUAUUCCACCUGGTT-3’ (antisense); the sequences for mouse JNK-siRNA were 5’-GGAGGAACGAACUAAGAAUTT-3’ (sense) and 5’-AUUCUUAGUUCGUUCCUCCTT-3’ (antisense); and the sequences for mouse NEK7-siRNA were 5’-GCAACUCAACCAUCCAAAUTT-3’ (sense) and 5’- AUUUGGAUGGUUGAGUUGCTT-3’ (antisense). Sequences for the NC-siRNA were 5’-UUCUCCGAACGUGUCACGUTT-3’ (sense) and 5’-ACGUGACACGUUC GGAGAATT-3’ (antisense). Following 48 h of transfection, the cells were infected with *S. aureus* and harvested for western blot or RT-PCR. The efficiency of Syk, JNK or NEK7 silencing was assessed by western blot and RT-PCR.

### Western Blot

Macrophages at a concentration of 1x10^6^ cells/well were infected with *S. aureus* at an MOI of 1 or 5. At the harvest time, cells were lysed by SDS lysis buffer and lung tissues were homogenized in RIPA buffer. Total protein concentration was assessed using a bicinchoninic acid protein assay kit (Pierce; Thermo Fisher Scientific, Inc.). Samples were separated by SDS-PAGE, and transferred to polyvinylidene difluoride (PVDF) membranes (Merck. Millipore). PVDF membranes were blocked with 5% nonfat milk or 5% bovine serum albumin (BSA) for 2 h at room temperature (RT). Membranes were then incubated with primary antibody overnight at 4°C. Following three washes with Tris-buffered saline/Tween 20 (TBST), the membranes were incubated with secondary antibodies at RT for 1 h. After being washed 3 times with TBST, the corresponding protein bands were detected using enhanced chemiluminescent HRP substrate (Millipore). β-actin was employed as a loading control. The results were analyzed using ImageJ software 6.0 (National Institutes of Health, Bethesda, MD, USA).

### Enzyme-Linked Immunosorbent Assay

Mouse IL-1β and IL-18 proteins released into the supernatants were measured using an ELISA. The ELISA kits for IL-1β (cat.no. EK201B/3-96) and IL-18 (cat no. EK2182-96T) were purchased from Multi Sciences (Lianke) Biotech, Co., Ltd. (Hangzhou, China).

### Coimmunoprecipitation

Macrophages at a concentration of 9x10^6^ cells/well were seeded in 10 cm plates and infected with *S. aureus* at an MOI of 1 for 24 h. Cells were lysed in cold lysis buffer (40 mM Tris base, 120 mM NaCl, 1% Triton X-100, 0.5 mM Na3VO4, 1 mM PMSF, 0.5 mM NaF, 1 × EDTA-free Roche protease inhibitor cocktail, pH 7.4) with shaking at a high speed for 30 min. Cell lysates were collected by centrifugation (12,000 × rpm) at 4°C for 30 min. Total protein concentrations were determined using a BCA protein assay. Some of the samples were used to detect the expression level of intracellular proteins. Dynabeads™ Protein A were incubated with anti-NEK7 (4 *μ*g) or control IgG (4 *μ*g) antibodies on a rotary shaker at 4°C for 6 h. The other samples were equalized into two tubes and incubated with Dynabeads carrying different antibodies by shaking at 4°C overnight. The beads were washed more than 5 times. Proteins were denatured at 97°C for 5 min following the addition of twofold loading buffer and then detected by western blot.

### Immunofluorescence Staining

J774A.1 cells (at a concentration of 2×10^5^/well) were seeded in 12-well plates on slides. Adherent cells were treated according to the experimental conditions. Slides were washed with PBS three times and fixed in 4% paraformaldehyde at RT for 15 minutes. Slides were blocked in 5% BSA buffer at 37°C for 1 h and incubated with anti-ASC antibody at 4°C overnight. After being washed three times with PBS, the cells were incubated with secondary antibody for 1 h at RT. DAPI was used to stain nuclei. Cell images were taken using a Nikon DS-Ri1 fluorescence microscope. The ASC specks in at least 5 random fields of one slide were counted, and the percentage of ASC-speck-positive cells was calculated.

### H&E Stain

The lung tissue of mice at 24 h postinfection was fixed in 4% paraformaldehyde and routinely processed into paraffin. Sections 5 *µ*m in thickness were cut for hematoxylin and eosin staining according to the manufacturer’s protocol for histopathologic evaluation. The histopathological samples were blindly assessed according to a histological scoring system as previously described (42). In short, on a scale of 0 to 4 for four pathological parameters were scored: (1) alveolar congestion, (2) hemorrhage, (3) inflammatory cell infiltration, and (4) alveolar wall thickness. 0 points represent normal lung, and scores of 1, 2, 3, and 4 represent mild (<25%), moderate (25%-50%), severe (50%-75%), and very severe (>75%) lung involvement, respectively. The total score was based on the sum of all score. Control group (1-3); infection group (4-6).

### RT-PCR

Total RNA was harvested using TRIzol reagent according to the manufacturer’s protocol, and first-strand cDNA was synthesized using the FastQuant RT kit (Tiangen Biotech Co., Ltd., Beijing, China). The concentration of the isolated RNA was measured using a NanoDrop (Thermo Fisher Scientific, Inc.). The mRNA levels were analyzed by RT-PCR using SYBR Green. The PCRs were performed with an initial denaturation at 95°C for 10 min, followed by 40 cycles at 95°C for 10 sec, 60°C for 20 sec, and 72°C for 20 sec, with a final extension at 72°C for 5 min. The PCR products were analyzed by melting curve analysis using MxPro-Mx3005P software v4.10 (Agilent Technologies, Inc.). The relative quantification of mRNA was performed using the comparative quantification cycle (Cq) method. The mRNA expression level was measured using the 2^−ΔΔCq^ method.

### Cell Toxicity Assay by LDH Release

Macrophages were seeded in 6-well-plates for 2.5 h and infected at an MOI of 1 for 24 h by culturing with or without KCl (40 mM). LDH release in supernatants was measured according to the manufacturer’s protocol using an LDH assay kit (cat. no. BC0685, Beijing Solarbio Science & Technology Co., Ltd.).

### Potassium Assay

Macrophages were seeded in 6-well-plates for 2.5 h and infected at an MOI of 1 for 24 h. The concentrations of intracellular and extracellular potassium were measured following the manufacturer’s protocol *via* a biochemical assay kit (cat. no. E-BC-K279-M, Elabscience Biotechnology Co., Ltd. Wuhan, China).

### Statistical Analysis

Data are presented as the means ± SEM deviation of independent experiments. All the statistical results are representative of three independent experiments, and differences between groups were analyzed by GraphPad Prism 8 software (GraphPad Software, Inc., La Jolla, CA, USA). Student’s t-test, one-way ANOVA with Tukey’s *post hoc* test, and two-way ANOVA with Tukey’s multiple comparisons test were used for analysis. A statistically significant difference was defined as *p*<0.05 (^*^ *p*< 0.05, ^**^ *p*< 0.01, ^***^ *p*< 0.001).

## Data Availability Statement

The original contributions presented in the study are included in the article/supplementary material. Further inquiries can be directed to the corresponding authors.

## Ethics Statement

The animal study was reviewed and approved by the Laboratory Animal Ethical Committee of Tianjin Medical University.

## Author Contributions

RL: data curation, project administration and writing-original draft. RL, YS, DT: conceptualization. RL, YL, AG, LW, HT: investigation. YS, ZQ, CL, XW: funding acquisition. CL, QW: supervision. All authors contributed to the article and approved the submitted version.

## Funding

This work was supported by grants of the National Natural Science Foundation of China to YS (81772252, 32071263) and ZQ (81971887, 82172170); the Tianjin Natural Science Foundation to ZQ (20JCYBJC01260, 20YDTPJC00250); the Key Laboratory of Emergency and Trauma (Hainan Medical University), Ministry of Education to YS (KLET-201906) and ZQ (KLET-201906); the Fundamental Research Funds for the Central Universities, Nankai University to ZQ (63211140); the Scientific Research Project of Tianjin Education Commission to CL (2020KJ206); the Science & Technology Department of Sichuan Province to XW (2020YFS0103).

## Conflict of Interest

The authors declare that the research was conducted in the absence of any commercial or financial relationships that could be construed as a potential conflict of interest.

## Publisher’s Note

All claims expressed in this article are solely those of the authors and do not necessarily represent those of their affiliated organizations, or those of the publisher, the editors and the reviewers. Any product that may be evaluated in this article, or claim that may be made by its manufacturer, is not guaranteed or endorsed by the publisher.

## Supplementary Material

Supplementary Figure 1 JNK and Syk activated the NLRP3 inflammasome upon *S. aureus* infection in PECs. PECs pretreated with or without JNK inhibitor at the final concentration of 20 μmol/L or Syk inhibitor at the final concentration of 1 μmol/L for 2 h followed by *S. aureus* infecton (MOI of 1) for 24 h. **(A)** The mRNA expression levels of *caspase-1*, *Il-1β* were quantified by RT-PCR. **(B)** The protein levels of caspase-1, p20, pro-IL-1β and mIL-1β were examined by western blot and analyzed by relative densitometric quantification. **(C)** Immunofluorescence imaged endogenous ASC specks. Cells were fixed and stained for ASC (Green), DAPI (Blue). (Scale bar: 100 µm.) **(D)** The mRNA expression levels of *caspase-1*, *Il-1β* were examined by RT-PCR. **(E)** The protein expression levels of caspase-1, p20, pro-IL-1β and mIL-1β were examined by western blot and analyzed by relative densitometric quantification. **(F)** The ASC specks formation were imaged *via* fluorescence microscopy. (Scale bar: 100 µm.). Similar results were obtained in three independent experiments in data. Data shown are means ± SEMs; ***P* < 0.01, *****P* < 0.0001 vs. non-infection control group, ^#^ *P* < 0.05, ^##^ *P* < 0.01, ^####^ *P* < 0.0001 *vs*. infection control group.

## Abbreviations

AIDS, acquired immune deficiency syndrome; ATP, adenosine triphosphate; ASC, apoptosis-associated speck-like protein containing a caspase-recruitment domain; BALF, bronchoalveolar lavage fluid; CFU, colony-forming units; DCM, diabetic cardiomyopathy; DN, diabetic nephropathy; H&E, hematoxylin and eosin; JNK, c-Jun N-terminal kinase; KCl, potassium chloride; IL-1β, interleukin-1β; IL-18, interleukin-18; mIL-1β, mature IL-1β; MRSA, methicillin-resistant *Staphylococcus aureus;* NEK7, NIMA-related kinase 7; NLRP3, NOD-like receptor family, pyrin domain containing 3; PAMPs, pathogen-associated molecular patterns; PECs, peritoneal exudate cells; p20, activated caspase-1; ROS, reactive oxygen species; RSV, resveratrol; SLE, systemic lupus erythematosus; *Staphylococcus aureus*, *S. aureus*; Syk, spleen tyrosine kinase.
