# Supplementary figures and images for: NEK7-Mediated Activation of NLRP3 Inflammasome Is Coordinated by Potassium Efflux/Syk/JNK Signaling During Staphylococcus aureus Infection
Source: Front Immunol. 2021 Sep 16;12:747370. doi: 10.3389/fimmu.2021.747370 (PMC8481599; doi:10.3389/fimmu.2021.747370)

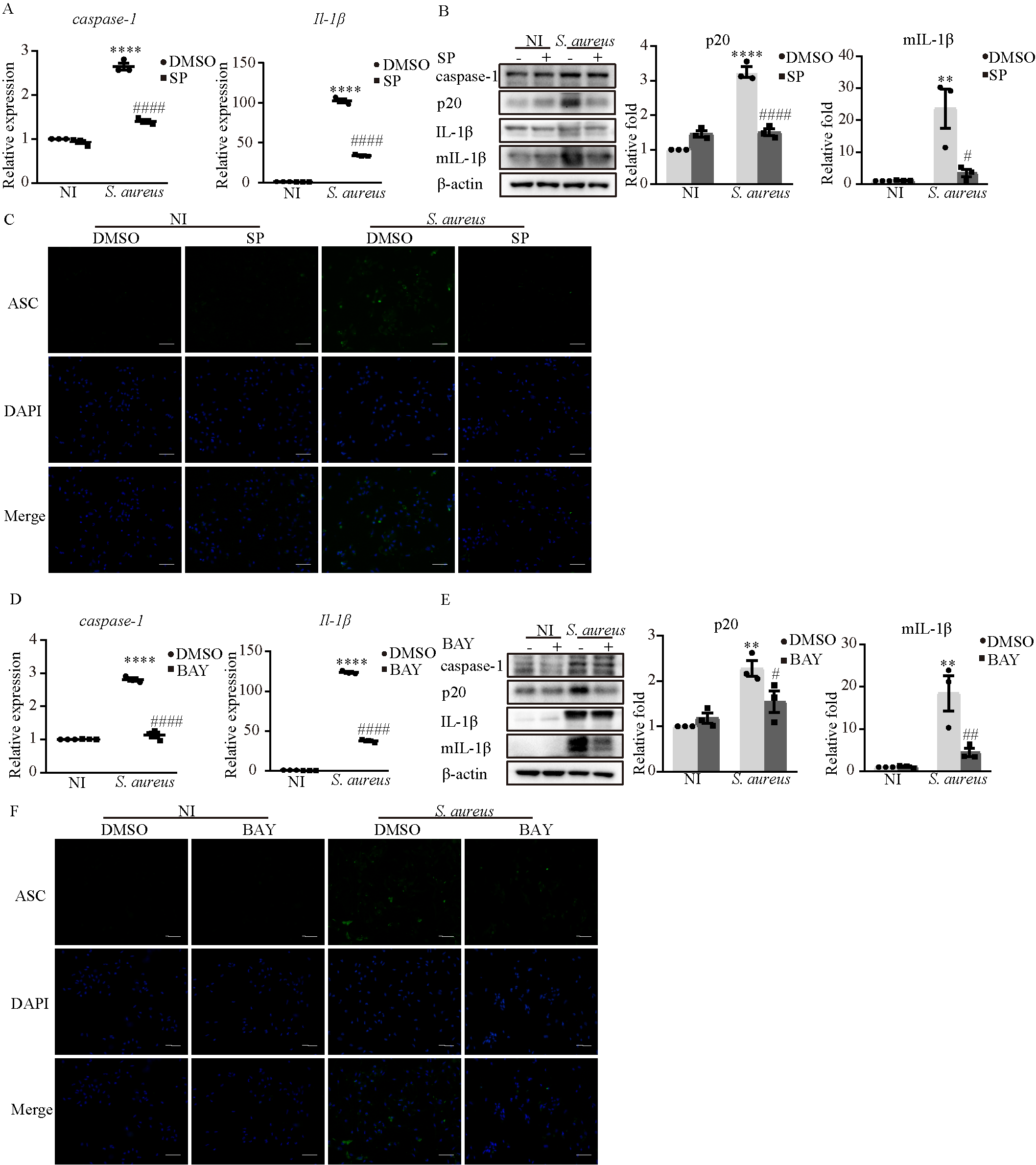

Supplement: Supplementary Figure 1 — JNK and Syk activated the NLRP3 inflammasome upon S. aureus infection in PECs. PECs pretreated with or without JNK inhibitor at the final concentration of 20 μmol/L or Syk inhibitor at the final concentration of 1 μmol/L for 2 h followed by S. aureus infecton (MOI of 1) for 24 h. (A) The mRNA expression levels of caspase-1, Il-1β were quantified by RT-PCR. (B) The protein levels of caspase-1, p20, pro-IL-1β and mIL-1β were examined by western blot and analyzed by relative densitometric quantification. (C) Immunofluorescence imaged endogenous ASC specks. Cells were fixed and stained for ASC (Green), DAPI (Blue). (Scale bar: 100 µm.) (D) The mRNA expression levels of caspase-1, Il-1β were examined by RT-PCR. (E) The protein expression levels of caspase-1, p20, pro-IL-1β and mIL-1β were examined by western blot and analyzed by relative densitometric quantification. (F) The ASC specks formation were imaged via fluorescence microscopy. (Scale bar: 100 µm.). Similar results were obtained in three independent experiments in data. Data shown are means ± SEMs; **P < 0.01, ****P < 0.0001 vs. non-infection control group, # P < 0.05, ## P < 0.01, #### P < 0.0001 vs. infection control group. [file Image_1.tif]
